# Social Capital and Its Returns as an Explanation for Early Labor Market Success of Majority and Minority Members in the Netherlands

**DOI:** 10.1007/s11205-022-03002-8

**Published:** 2022-09-24

**Authors:** Ids Baalbergen, Eva Jaspers

**Affiliations:** 1 Department of Human Geography and Spatial Planning, Utrecht University, Utrecht, The Netherlands; 2 Department of Sociology and ICS (Inter-University Centre for Social Science Theory and Methodology), Utrecht University, Utrecht, The Netherlands

**Keywords:** Social capital, Return deficit, Immigrants, Labor market inequality, Youth unemployment, The Netherlands

## Abstract

This paper tests whether social capital can explain differences in labor market success between ethnic majority and minority members. To overcome problems of reverse causality—labor market success is not only the result of social capital, but also leads to better networks—the focus is on adolescents who enter the labor market. Data from the ‘Children of Immigrants Longitudinal Survey’ are used (N = 2574) and matched to register data from Statistics Netherlands. Hypotheses are tested with structural equation models and a longitudinal approach. Two different mechanisms are tested: the capital deficit and the return deficit. Ethnic majority and minority members do not differ in social capital, thus refuting the capital deficit hypothesis. However, for majority members, the upper reachability of their social capital negatively affects chances of unemployment and positively affects chances of having a permanent contract. For minority members, no such effects were observed, indicating that the same level of social capital that benefits majorities, does not benefit minorities. More research into the return deficit minority members face is needed.

## Introduction

In Europe, second-generation immigrants with origins in the Global South^1^ occupy a disadvantaged position when entering the labor market. During the peak of the economic crisis in 2014, their youth unemployment rate was 1.5 times higher than the youth unemployment rate among those who were native-born and whose parents were also native-born (Eurostat, 2016a). Furthermore, (young) ethnic minorities are more likely to have a temporary contract or work part-time (Eurostat, 2016b) and they less often occupy professional and managerial jobs in several European countries (Heath et al., 2008). Differences in early labor market success are particularly influential since such differences may lead to cumulative disadvantages (Arulampalam et al., 2001; Gregg, 2001; Petersen et al., 2011). A lack of early labor market success among minority members may thus disproportionately affect their careers. Previous research has found that this is especially the case for early-career unemployment. Experiencing unemployment early on may have a scarring effect on someone’s career (Arulampalam et al., 2001; Gregg, 2001).

The relevance of one’s social network in explaining ethnic differentials in initial labor market success has been underlined in previous research (Verhaeghe et al., 2015). Members of an individual’s social network have resources that they can employ on behalf of said individual (Völker & Flap, 1999). According to the 1996 Eurobarometer, 38% of the unemployed search for a job through family and 63% search for a job through friends (Gallie, 1997). Holzer (1987) found that 85% of the unemployed youth in the United States asked friends or relatives about possible job openings. Pellizzari (2010) showed that 15% of the employed workers in the United States found their current job via personal contacts; the average in fourteen European countries is around 20%. Furthermore, in several countries, there is a positive relationship between knowing people in prestigious occupations and labor market success (Behtoui, 2007; Li et al., 2008; Völker & Flap, 1999). Verhaeghe et al. (2015) show ethnic inequalities in social capital in Belgium, which they can attribute mostly to class differences between minorities and majorities.

Relatively little is known about the role of networks in explaining ethnic differences in labor market outcomes, and what is known, is potentially confounded. As labor market success in turn leads to larger and better networks (Mouw, 2003), problems of reverse causality occur when analyzing associations between social capital and labor market outcomes. By focusing on youths of the same grade cohort, at the start of their labor market careers, we can overcome this issue.

In this paper, we will therefore study whether differences in social capital between majority and minority members can explain early labor market inequalities in the Netherlands. Our aim is threefold. One, we want to establish whether such ethnic inequalities in the social capital of young people exist in the Netherlands. Second, we investigate whether the social capital of adolescents themselves affects early labor market success after controlling for the socioeconomic background of their parents. Third, we wish to establish whether ethnic minority youth in the Netherlands face a lack of access to social capital, or rather lower returns on their existing social capital (Song & Lin, 2009; Pedulla & Pager, 2019).

Since social capital has many definitions and conceptualizations (Van Tubergen & Völker, 2015), it is necessary to clarify our understanding of adolescent social capital in this paper. We follow previous research which focused on the socio-economic resources that are embedded in people’s network that can be accessed and mobilized (Lin, 2001). Social capital thus consists of (1) the people an individual knows; (2) the resources they possess; and (3) their willingness and opportunities to employ these resources for said individual (Pedulla & Pager, 2019). The resourcefulness of one’s network is a combination of the three components (Van Tubergen & Völker, 2015). Like other forms of capital (e.g., human capital), social capital can be seen as a resource that can be used to gain returns in instrumental actions such as finding a job (Lin, 1999a). Ethnic differences in either the available resources of their social network members, or the willingness of their network members to employ these resources for said minorities, can thus result in ethnic differences in labor market success.

Evidence for a lower level of social capital among minority members has been found in several countries such as Sweden (Behtoui, 2007), the United Kingdom (Li et al., 2008), and the United States (McDonald, 2011). In the Netherlands Van Tubergen and Völker (2015) found evidence for it among people with a Moroccan immigration background, but not among people with a Turkish immigration background. Verhaeghe et al. (2015) observed that ethnic differentials in social capital can be explained by social class origins in Belgium. Moreover, Behtoui (2007) and McDonald (2011) found that differences in social capital between minority and majority members can partially explain differences in labor market success. Lancee (2010, 2012) showed that contacts with native backgrounds, lead to improved labor market outcomes for ethnic minorities in the Netherlands and Germany. Few studies have addressed ethnic differentials in the returns to social capital in Europe. However, Lancee (2016) showed that job changes were more beneficial for higher educated immigrants with more native German friends, indicating that they receive at least some returns on their social capital. Our research question thus reads:

Can ethnic differences in social capital explain differences in early labor market success of majority and minority members in the Netherlands?

Next to our three aims, the current study improves upon previous work by using novel and multiple measures of the social capital of adolescents themselves, which allows us to study different dimensions of social capital simultaneously. We will test whether the social networks of minority and majority members differ in the number of connections to prestigious individuals, the diversity of their relations, and the extensity or range of their resources- together the quality of their social capital; and whether they receive different levels of support from their social networks—the accessibility and returns part of their social capital.

Previous research has offered several explanations for differences in labor market success between minority and majority members that we will not address explicitly in this paper. We will, however, control for the, on average, lower human and cultural capital of minorities (Aschaffenburg & Maas, 1997; Corak, 2013; Oaxaca & Ransom, 1994; Van Ours & Veenman, 2004), as well as for the socio-economic background of the parents (Boudon, 1974; Breen & Jonsson, 2005; Van Ours & Veenman, 2003). Lastly, labor market discrimination of minorities may also be detrimental to their labor market success (Blommaert et al., 2013; Heath et al., 2008; Reimers, 1983). In the current paper, we cannot control for this latter explanation, but we argue that this should be observed in lower levels of success per se, not in the effect of social capital on early labor market success.

We will be using data from the ‘Children of Immigrants Longitudinal Survey in Four European Countries’ (CILS4EU) (Kalter et al., 2016a, 2016b, 2016c), and the ‘Children of Immigrants Longitudinal Survey in the Netherlands’ (CILSNL) (Jaspers & Van Tubergen, 2014, 2015, 2016, 2017), which is the Dutch continuation of the CILS4EU project. The CILS data is especially suitable for the current study because it focuses on youth from a school grade cohort and includes multiple measurements of social capital. Furthermore, minorities are oversampled in the panel data, increasing the number of minority members in our study. Respondents were followed from adolescence to early adulthood and matched to labor market information from Statistics Netherlands. Studying the effect of social capital on early labor market success is a particularly useful endeavor because possible confounding factors, such as human capital attained during employment, can -partially- be excluded.

## Theory

**Fig. 1:**
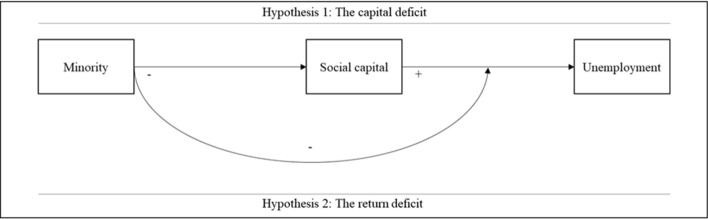
Schematic overview of the capital and return deficit

The theory section will be structured as follows. First, the role of social capital in the labor market is discussed. Second, we will discuss possible explanations for why minority members may have lower quantity and/or quality social capital than majority members, referred to as *the capital deficit* (Lin, 2000). Third, arguments about differences in accessibility of social capital between minority and majority members are considered, which is labeled *the return deficit* (Lin, 2000). Figure 1 gives a schematic overview of the two deficits.

### Social Capital and Labor Market Success

It has been argued that social capital may be useful in the labor market for both minorities and majorities. Lin (1999b) proposes four mechanisms through which social capital influences labor market outcomes. Firstly, social contacts may provide access to labor market information, such as jobs, and being better informed about job-openings may subsequently lead to additional opportunities in the labor market (Granovetter, 1973). Secondly, social contacts may influence others in their decision-making, a social contact may for instance vouch for an individual during a hiring process (Marsden, 1994). Thirdly, the social capital of an individual may be considered a resource in itself for an organization, therefore individuals with more social capital may be more desirable employees (Erickson, 2001). Lastly, social contacts may provide emotional support, which may aid them in locating jobs and buffers them from the stress of unemployment (Caspi et al., 1998). We will refrain from arguing about this fourth explanation, as it is outside the scope of our paper.

Previous research has criticized the assumption that just being connected to people in higher socioeconomic positions is always most beneficial. Ruiter and De Graaf (2009) argued that being connected to people in similar socioeconomic positions may be more beneficial for an individual than being connected to people in higher socioeconomic positions. People in similar occupations may be able to provide more relevant information. A carpenter may, for example, be more likely to receive useful information from a foreman in construction than from a lawyer, even though the latter occupies a higher socioeconomic position. For that reason, only focusing on connections with people in prestigious socioeconomic positions may be too simplistic.

Other research has argued that there are three dimensions of social capital (Behtoui, 2007; Song & Lin, 2009), namely: upper reachability, extensity, and range. Upper reachability indicates the extent to which an individual has access to prestigious occupations. Extensity estimates the quantity of one’s social capital. Finally, range reflects the diversity of one’s social capital. Differentiating between these dimensions may help in capturing the concept of social capital more accurately, as high scores on all three dimensions carry unique advantages (Song & Lin, 2009).

### The Capital Deficit

Previous research has investigated whether majority and minority members differ in the amount of social capital they possess. These studies investigated whether the networks of majority and minority members differ in size and resourcefulness, the first two components of social capital (Lancee, 2016; Van Tubergen & Völker, 2015). Evidence for a social capital deficit among minority members has been found in several countries (Behtoui, 2007; Li et al., 2008; McDonald, 2011; Van Tubergen & Völker, 2015).

A possible explanation for the observed capital deficit was given by Lin (2000). People tend to have more contact with people who are similar to them than with people who are dissimilar to them in key demographic characteristics (McPherson et al., 2001). This phenomenon is also known as ‘homophily’. Homophily along ethnic and racial lines may impede contact between majority and minority members. This homophily principle may hinder the social capital of minorities because on average minorities have lower socioeconomic status than majority members. Contact with people higher in socioeconomic status may be more beneficial to one’s social capital than contact with people lower in socioeconomic status. People higher in socioeconomic status may possess more social resources (Lin et al., 1981), that could be employed for others. Homophily along ethnic lines may then make it difficult for minorities to establish or maintain the same quality of their networks as majority members.

It has previously been found that minority members have not only less prestigious, but also smaller and less diverse social networks (Li et al., 2008; Marsden, 1987). Behtoui (2007) argues that minority members are more likely to live in stigmatized neighborhoods or work in inferior parts of the labor market. This may hinder their opportunities of meeting different kinds of people, which in turn may impact network size and diversity. Parents of minorities may also have less socioeconomic resources on average, while such parental resources may also lead to social capital (Lai et al., 1998). Parental social capital might be particularly important for young people’s early labor market success, when their own social capital is still in its developmental stages.

Based on the previous, we expect a social capital deficit among minority members affecting their labor market outcomes. Therefore, the following hypothesis is proposed:

#### **Hypothesis 1**

Ethnic minority members have a social capital deficit compared to majority members, and therefore they have less early labor market success.

### The Return Deficit

While a capital deficit implies that members of different social groups differ in the quantity or quality of social capital they possess, a return deficit implies that members of different social groups may receive different returns on the same social capital. Thus, majority and minority members who have networks similar in size and social resources may still receive different returns. This is related to the third component of social capital: differences in the willingness and opportunities of one’s contacts to employ their resources for said individual (Van Tubergen & Völker, 2015). Scholars have stated that such a deficit has only scarcely been tested (Lin, 2000; Pedulla & Pager, 2019). Smith (2005) did, however, find evidence for a return deficit among African-Americans. She found that poor, urban African-Americans were less likely to benefit from their relations, not because they lacked social capital but because their social capital was less likely to be mobilized for assistance.

The social capital of minority members may less often be mobilized because their contacts are less often willing to provide help compared to the contacts of majority members. Majority members may view other majority members as more competent, and may for that reason prefer to provide network resources to them instead of minority members (McDonald, 2011). The returns of belonging to a network of majority members high in socioeconomic status may then be smaller for minority members than for majority members. A related mechanism was discussed by Smith (2005), who argued that the decision to provide job-finding assistance is dependent on both one’s own reputation and the job seekers’ reputation. Recommending someone can potentially damage one’s reputation. She found that poor urban African Americans had negative perceptions about the work ethic and motivation of other poor urban African Americans. Due to those perceptions, they were less willing to provide referrals to them because they did not want to risk their reputation. Ethnic minorities in the Netherlands may reap fewer returns from their social capital if similar processes operate for them. Alternatively, the perception that employers are more hesitant to employ minorities may prevail among the contacts of minorities, which prevents them from referring said minorities out of reluctance to offer unappreciated suggestions (Lin, 2000). The perception that referring a minority is unlikely to be valued may thus further hinder contacts from referring minorities.

There may also be a lack of opportunity among the contacts of minorities to successfully provide help (Van Tubergen & Völker, 2015). Receiving a referral may be less beneficial to minority members than to majority members if employers discriminate against minorities after referrals, and for that reason, less often hire them (Heath et al., 2008). Employers could thus respond differently to minority members than to majority members, even if they have equal amounts of social capital. Mobilizing social capital would be more beneficial for majority members, than for minority members. Holzer (1987) further argued that employers may have more confidence in the referrals that currently employed majority members give compared to referrals that currently employed minority members give. Due to homophily, the networks of minorities may to a large extent consist of other minorities. They may, therefore, mainly receive referrals from other minorities who have fewer opportunities to successfully provide help. Mobilization of an employed individual from the same ethnic group may, in that case, be more beneficial for majority members than for minority members.

Based on the previous arguments, the following hypothesis concerning the return deficit is proposed:

#### **Hypothesis 2**

The returns to social capital are lower for minority members than for majority members, and therefore they have less early labor market success.

## Data and Method

### Data

The role of social capital in the early-career labor market success of minority and majority members is studied with panel data from the CILSNL and CILS4EU projects, and register data from Statistics Netherlands. The CILSNL project is the Dutch continuation of the CILS4EU project. The CILS4EU panel was requested to participate in CILSNL as well. The CILS4EU project consisted of three waves and was collected between 2010 and 2013. Respondents were approximately 14 years old in the first wave. The CILSNL project consisted of four additional waves and was collected between 2013 and 2017. In the latest wave of data collection, respondents were approximately 21 years old. After merging all the waves of panel data, they were linked to register data from Statistics Netherlands.

The initial sample of the CILS4EU project was drawn via a three-stage sampling design where individuals were selected within schools and school classes. Schools were selected with probability proportionally to size. Furthermore, schools with larger proportions of immigrants were more likely to be selected to acquire data about a large enough number of students with an immigration background. These schools are typically located in more urban areas, within somewhat lower socio-economic neighborhoods, which means that the study is selective in these respects. Schools that refused to participate were replaced by schools with similar characteristics, resulting in a response rate of 91.7% at the school level. After schools had agreed to participate, two school classes were randomly selected for participation. The response rate at the class level was 94.5%. Within these school classes, 91.1% of the students participated. The sampling procedure aimed to enable inferences for Dutch adolescents of one cohort of students from the same grade in secondary education (CILS4EU, 2016). However, given the complex sampling and replacement strategy of the panel, the data cannot and should not be interpreted as representative for the entire Dutch population of 14 years old adolescents. It is possible though, to identify mechanisms for this cohort.

In the current study, the CILS4EU data provide information about social capital, immigration background, occupational status of parents, age, gender, and enable clustering observations within schools. The CILSNL data contain additional measures of social capital. Lastly, the register data provide information about early-career labor market success, such as unemployment spells and precariousness of jobs, and level of education.

#### Selection

In total, 7,331 participated in any of the CILS4EU waves. Part of the respondents were excluded from the analysis because they could not be asked for permission to link their panel data to register data (N = 2.669) as they had already dropped from the panel prior to the linkages were made, did not give permission or could not be linked to the panel data (N = 158), or had missing data on a clustering variable (N = 17). In addition, 1,913 respondents were left out of the analysis because they were still studying at the latest wave of available register data (December 2019). For that reason, their early-career labor market success could not be studied. The final sample consists of 2574 respondents, of which 498 respondents with a minority background.

Due to the selection of cases, a relatively large share of the initial panel was excluded from the analysis. This has resulted in a loss of statistical power and may have caused bias. Bias patterns were investigated through a logistic regression analysis (see Appendix 1). The analysis showed that male respondents and respondents with a minority background were more likely to attrite. This also held for respondents with a higher level of education and respondents with parents in more socioeconomically prestigious occupations.

Bias might partially be countered by postponing the analysis until more respondents finished studying. However, after the COVID-19 pandemic started in February 2020, youth unemployment rose from 6.3 to 11.3% in August 2020 (Statistics Netherlands, 2021). Additionally, the temporary emergency scheme for job retention that the government implemented may have influenced who became unemployed. Therefore, comparing pre-pandemic labor-market success with labor-market success after the pandemic started is problematic. We believe that restricting our analysis to early-career labor-market success before the COVID-19 crisis started is an apt solution.

### Measures

#### Dependent Variable

The current study measures early labor market success through two dependent variables. The first dependent variable is early-career unemployment. Respondents who received unemployment benefits or social assistance benefits in the first twelve months after finishing education^2^ were classified as unemployed (1 = *unemployed*, 0 = *not unemployed*). Respondents who were not employed but did not receive benefits were classified as not unemployed to exclude respondents who were voluntarily not working from the unemployed category. Similarly, respondents who received disability benefits were classified as not-unemployed. The second dependent variable is early career job-security. This is defined as a job with a permanent contract for at least 32 h per week within eighteen months after graduating (1 = *secure job*, 0 = *insecure job*). The period of eighteen months was chosen because in the Netherlands, employees are often offered a temporary contract in the first year of employment.

#### Independent Variables

The current study investigates differences between respondents with and without an immigration background in the Global South (Dutch: non-western immigration background). Following the initial definition in the CILS4EU sample, persons who are themselves, or have at least one parent, born in most countries in the Global South are considered as having an immigration background in the Global South (CILS4EU, 2016). According to Statistics Netherlands (2022), countries in the Global South are countries in Africa, Latin America, Asia (excluding Indonesia and Japan), and Turkey. A dummy variable was constructed based on this definition (1 = *minority*, 0 = *majority*).

It has previously been argued that social capital has three dimensions: extensity, upper reachability, and range (Li et al, 2008; Song & Lin, 2009). These dimensions have in the past been studied using position generator measures. In a position generator, respondents indicate whether they know people who meet particular characteristics (Lin & Dumin, 1986). Most studies have used lists of occupations differing in prestige. Respondents were subsequently asked whether or not they knew someone in each of the occupations (e.g., Behtoui, 2007; Li et al., 2008; Van Tubergen & Völker, 2015). However, the age of the respondents in the current study would make that approach problematic. The respondents were followed from adolescence into early adulthood. During this period, the social world of respondents may to a large extent consist of people who are not yet employed. A novel approach was used because people who are not employed may also provide useful social resources (Van der Gaag & Snijders, 2005). Multiple measures collected across all seven waves were combined to construct two reflexive latent variables, namely, upper reachability and range, and one observed variable related to network extensity. An overview of all the items that were used to measure social capital can be found in Appendix 2.

Network extensity is about the size of one’s network. During wave four, respondents indicated how many people they know with particular names and how many people they know who live in particular cities, a so-called scale-up method for estimating network size (McCormick et al., 2010). Seven response categories were used: 0, 1, 2–5, 6–10, 11–20, 21–50, *and more than* 50. Mean scores were computed based on the categories. Thereafter, the mean scores were divided by the total number of people with a specific name in the Netherlands or by the total number of people living in a particular city. Then, the number was multiplied by the total population of the Netherlands. After that, the mean over the ten scores was taken, and lastly, the natural logarithm was calculated.

Upper reachability was measured based on contact with people in prestigious social positions and the average level of education of one’s friends. In wave six, respondents indicated how many people they know who are a lawyer, a professor, or own a villa. Again, seven response categories were used: 0, 1, 2–5, 6–10, 11–20, 21–50,* and more than* 50. Mean scores were computed based on the categories. During the last four waves, respondents were asked about the highest level of education their three best friends attended or were currently attending. Five categories were used (1 = *lower secondary*, 2 = *higher secondary*, 3 = *lower vocational*, 4 = *higher vocational, and *5 = *university*). Based on the level of education across all friends and waves, a mean score was constructed. Thus, upper reachability was estimated by three variables related to contact with people in prestigious social positions and one variable related to the average level of education of one’s friends.

Lastly, range was measured based on contact with people with different immigration backgrounds, having friends with different immigration backgrounds, and knowing people at different levels of education. During the second wave, respondents were asked how often they talked to people with a Dutch, Moroccan, Surinamese/Antillean, Turkish, or another background. Five response categories were used (1 = *never*, 2 = *less often than monthly*, 3 = *once or several times a month*, 4 = *once or several times a week*, 5 = *every day)*. A mean score was constructed based on the five backgrounds. In all waves, respondents were asked about the background of their friends. They were asked whether they have friends with a Dutch, Moroccan, Surinamese/Antillean, Turkish, or another background. A dummy variable was constructed (0 = *none or very few*, 1 = *a few or more*). After that, a measure for friends’ diversity in each wave was constructed by summing the dummies (ranging from 0 to 5), which was used to construct a mean friend diversity score over all waves. The last aspect of range is related to knowing people at different levels of education. In wave four, respondents were asked how many people they know who attend vocational education, applied university, and university. The variables were recoded into three dummy variables (0 = *no one*, 1 = *one or more*). After that, the three dummies were summed.

#### Control Variables

Several control variables are included in the analysis. First, we will control for the highest attained level of education. The variable was recoded into six categories: lower secondary education, higher secondary education, lower vocational education, higher vocational education, applied university, and university. We will also control for the socioeconomic background of respondents. In the first wave, respondents named the occupations of both their parents. The occupation with the highest ISEI is included, which is a source of ascribed social capital in itself (Vacchiano et al., 2021). Furthermore, a dummy variable about the gender of respondents (0 = *female*, 1 = *male*), and a continuous variable about the age of respondents are included.

### Analytical Strategy

The sampling of CILS4EU was done at the school level. For that reason, we account for school-level clustering through cluster robust-standard errors. The hypotheses are tested using two sets of structural equation models with early-career unemployment and job security as dependent variables. The use of register data allowed us to measure social capital at an earlier time point than labor market success.^3^ All control variables are included in each model. Missing data are handled with full information maximum likelihood. A null model is estimated to assess model fit improvement.

The first hypothesis is tested by investigating whether minorities have less extensity, upper reachability, and range than majority members and whether the likelihood of unemployment / job security is mediated by differences in their social capital. The hypothesis can be confirmed if there is a significant indirect effect of immigration background on the likelihood of unemployment / job security through the three dimensions of social capital. The second hypothesis is tested by estimating the effect of the three dimensions of social capital on the likelihood of unemployment / job security in a separate model for majority and minority members. All parameters in the two models are constrained to be equal except for the effect of extensity, upper reachability, and range on the likelihood of unemployment / job security. The hypothesis can be confirmed if the three dimensions of social capital decrease the likelihood of unemployment / increase the likelihood of job security among majority members to a larger extent than among minority members. This will be assessed using Wald tests.

## Results

### Descriptive Statistics

Table 1 gives a bivariate overview of all descriptive statistics. Several aspects stand out. Minority members more often (*p* =  < .001) experience unemployment (11.5%) than majority members (5.0%). Minority members are also less likely (*p* =  < .001) to have a secure job (25.3%) than majority members (35.8%). Furthermore, the two groups differ in socioeconomic background. On average, majority members have a higher level of education than minority members. The ISEI of the parents of majority members is higher than the ISEI of the parents of minority members. Lastly, the share of men is smaller among minority members than among the majority members.

**Table 1:** Bivariate descriptive statistics (N = 2574)

	Majority members (N = 2076)		Minority members (N = 498)	
	Mean (%)	SD	Mean (%)	SD
*Dependent variables*				
Unemployed within 3 months	2.8%		6.8%	
Unemployed within 12 months	5.0%		11.5%	
Full time permanent contract	35.8%		25.3%	
*Independent variables*				
Social capital				
Upper reachability^a^	.0		.0	(.0)
Range^a^	.0		.1	(.0)
Extensity	1.8	(1.0)	2.3	(1.2)
*Control variables*				
Education				
Lower secondary	5.9%		12.1%	
Higher secondary	8.9%		8.8%	
Lower vocational	21.2%		25.9%	
Higher vocational	30.7%		27.8%	
Applied university	26.4%		20.7%	
University	6.9%		4.6%	
Parental ISEI	51.0	19.2	43.5	19.9
Age	23.6	.7	23.8	.8
Gender				
Male	41.7%		39.0%	
Female	58.3%		61.0%	

Percentages, means, and standard deviations for all items

^a^Under scalar invariance, the mean of the latent variable is 0 in the first group (majority members), and a free parameter in the second group

#### Confirmatory Factor Analysis

An overview of all the items that were used to measure social capital can be found in Appendix 2. Two reflective latent variables were used to measure upper reachability and range. The fit of the measurement model is mediocre^4^ (RMSEA = 0.073, CFI = 0.815, TLI = 0.647, χ^2^ = 838.669, *df* = 21, *p* =  < .001).

#### Structural Equation Models

Two sets of three structural equation models are estimated to test our hypotheses. Tables 2 and 3 summarize the models with early-career unemployment and job security as dependent variables respectively. Model 0 is used as the baseline model which is compared to the other models. The measurement model is included in this model and the likelihood of unemployment / job security is predicted by all independent and control variables. Immigration background and the three dimensions of social capital do not significantly affect the likelihood of unemployment / job security. A higher level of education decreases the likelihood of being unemployed while age increases the likelihood. None of the control variables had a significant effect on job security. The unemployment model explains 19% of the variance, but the fit of the model is mediocre (RMSEA = .064, CFI = .765, TLI = .733). The job security model explains less variance (7.1%) and the fit is similar (RMSEA = .064, CFI = .867, TLI = .737).

**Table 2:** Overview of structural equation models with unemployment as dependent variable (N = 2574)

	Model 0		Model 1		Model 2 majority		Model 2 minority	
	B	*SE*	B	*SE*	B	*SE*	B	*SE*
Threshold	3.927*	1.334	3.729*	(1.218)	5.248**	(1.439)	6.487**	(2.133)
*Independent variables*								
Minority	− .150	(.729)	− .944	(3.022)	–	–	–	–
Upper reachability	− .832	(.779)	− 1.228*	(.519)	− 2.937*	(1.273)	.673	(.726)
Range	2.813	(4.008)	5.013	(7.647)	1.065	(1.489)	6.644	(5.606)
Extensity	− .207	(.179)	− 0.167*	(.053)	− .131	(.134)	− .876	(.667)
*Indirect effects*								
Minority → upper reachability			.108**	(.020)				
Minority → range			.408**	(.066)				
Minority → extensity			1.713**	(.147)				
Minority → upper reachability → unemployed	− .132*	(.056)						
Minority → range → unemployed		2.045	(3.061)					
Minority → extensity → unemployed		− .286*	(.101)					
*Control variables*								
Education	− .207**	(.057)	− .224**	(.034)	− 0.234**	(.048)	− .234**	(.048)
Parental ISEI	− .522	(.295)	− .421	(.218)	− .325	(.358)	− .325	(.358)
Age	.165*	(.055)	.145*	(.051)	.210**	(.060)	.210**	(.060)
Male	− .102	(.083)	− .113	(.083)	− .149	(.103)	− .149	(.103)
*Model fit*								
RMSEA	.064		.061		.061		.061	
CFI	.765		.840		.886		.886	
TLI	.733		.762		.815		.815	
R^2^	.190		.168		.229		.036	

The dependent variable is unemployment within twelve months after graduation. All independent, and control variables are allowed to correlate. The loadings of the control variables in model 2a and model 2b are constrained to be equal

*p < .05; **p < .001

**Table 3:** Overview of structural equation models with job security as dependent variable (N = 2574)

	Model 0		Model 1		Model 2 majority		Model 2 minority	
	B	*SE*	B	*SE*	B	*SE*	B	*SE*
Threshold	− 0.117	.945	− .059	(.885)	0.106	(1.040)	0.554	(1.315)
*Independent variables*								
Minority	.377	(.888)	− .534	(.800)	–	–	–	–
Upper reachability	.811	(.856)	.676	(.352)	1.667	(.918)	− .001	(.394)
Range	− 3.240	(4.817)	− .014	(2.173)	− 1.835	(1.492)	2.250	(2.730)
Extensity	.154	(.212)	.052	(.035)	.153	(.129)	− .250	(.330)
*Indirect effects*								
Minority → upper reachability			.106**	(.020)				
Minority → range			.409**	(.066)				
Minority → extensity			1.712**	(.147)				
Minority → upper reachability → unemployed	.072	(.037)						
Minority → range → unemployed		− .006	(.890)					
Minority → extensity → unemployed		.088	(.062)					
*Control variables*								
Education	.101	(.054)	.130**	(.023)	.102*	(.036)	.102*	(.036)
Parental ISEI	− .030	(.289)	− .153	(.152)	.121	(.292)	.121	(.292)
Age	− .035	(.046)	− .020	(.037)	− .024	(.043)	− .024	(.043)
Male	− .073	(.064)	− .074	(.055)	− .081	(.063)	− .081	(.063)
*Model fit*								
RMSEA	.064		.060		.060		.060	
CFI	.867		.843		.887		.887	
TLI	.737		.765		.817		.817	
R^2^	.071		.056		.072		.054	

The dependent variable is job security within eighteen months after graduation. All independent, and control variables are allowed to correlate. The loadings of the control variables in model 2a and model 2b are constrained to be equal

*p < .05; **p < .001

#### The Capital Deficit

The first hypothesis is tested in model 1. Model 1 builds on the baseline model by adding immigration background as a predictor for upper reachability, range, and extensity. Furthermore, an indirect effect of immigration background on the likelihood of unemployment / job security through the three dimensions of social capital is included. As opposed to hypothesis 1, minority members have higher levels of social capital than majority members. They score higher on upper reachability, range, and extensity. The p-value of the indirect effect of immigration background on unemployment through extensity is below the threshold value, however, the effect is not in the expected direction. This is also the case for the indirect effect of upper reachability. None of the indirect effects on job security are significant. Thus, the first hypothesis is rejected.

Similar to the baseline model, higher levels of education decrease the likelihood of unemployment, while age increases the likelihood. In contrast to the previous model, higher levels of education increase the likelihood of job security. The model fit of the unemployment model (RMSEA = .061, CFI = .840, TLI = .762) and the job security model (RMSEA = .060, CFI = .843, TLI = .765) are both similar to the baseline models. The percentage of explained variance is smaller, 16.8% in the unemployment model and 5.6% in the job security model.

#### The Return Deficit

The second model builds on the baseline model by estimating the effect of the three dimensions of social capital in separate models for majority and minority members. In line with the second hypothesis, upper reachability decreases the likelihood of unemployment for majority members, while upper reachability does not significantly affect the likelihood of unemployment among minority members. A one-sided Wald test shows that the difference between the two groups is significant (χ^2^(1) = 6.232, *p* = .006). On the other hand, the other two dimensions of social capital (range and extensity) do not significantly affect the likelihood of unemployment in both groups. The job security model led to similar findings. While upper reachability does not have a significant main effect for either of the groups, a one-sided Wald test shows that the effect of upper reachability on unemployment does significantly differ between majority and minority members (χ^2^(1) = 3.648, *p* = .032). Therefore, the second hypothesis can partially be confirmed.^5^

In accordance with the previous model, higher levels of education decrease the likelihood of unemployment while age increases the likelihood. Similarly, education increased the likelihood of job security. The fit of both the unemployment model (RMSEA = .061, CFI = .886, TLI = 0.815) and job security model (RMSEA = .060, CFI = .887, TLI = .817) are comparable to previous models. In the unemployment model, the explained variance for majority members is comparatively high (22.9%) and comparatively small for minority members (3.6%). Similarly, more variance is explained for majority members (7.2%) than for minority members (5.4%) in the job security model.

## Discussion

In Europe, minority members occupy a disadvantaged labor market position (Eurostat, 2016a, 2016b; Heath et al., 2008). Several explanations for this disadvantage among minorities have been proposed, such as a lack of human or cultural capital (Aschaffenburg & Maas, 1997; Corak, 2013; Oaxaca & Ransom, 1994; Van Ours & Veenman, 2004), a lower socioeconomic background (Boudon, 1974; Breen & Jonsson, 2005; Van Ours & Veenman, 2003), and discrimination in the labor market (Blommaert et al., 2013; Heath et al., 2008; Reimers, 1983). The current study investigated whether social capital can explain differences in early-career labor market success between minority and majority members. Two mechanisms were studied: the capital deficit and the return deficit.

Partial evidence for the return deficit was found. Knowing people in prestigious social positions decreased the likelihood of early-career unemployment and increased the likelihood of early-career job security for majority members but not for minority members. So having high status others in the network is only beneficial to majority members. A larger or more diverse network did not affect majority and minority members differently. The current findings are important in the understanding of the role of social capital in the labor market. Although previous research has theorized about the return deficit, evidence has been lacking so far, as Lin already suggested in 2000 and as has recently been repeated by Pedulla and Pager (2019). The existence of a return deficit would have significant implications. Extending the social network of minorities through, for example, social mixing (Bolt et al., 2010) may be an ineffective measure when minorities are not able to reap the returns. The present study does not differentiate between the different mechanisms that could explain the observed effect, which future research should aim to understand. Furthermore, we were unable to account for the possibility that (part of) the social capital reported by minorities is located in a different country, which would make resources less valuable on the Dutch labor market. Future studies are encouraged to disentangle this.

Contrary to previous studies, minority members were found to have higher levels of social capital than majority members on our novel measures. In the Netherlands, one previous study also did not find differences between majority and minority members (Van Tubergen & Völker, 2015). Studies in other countries found higher levels of social capital among majority members (Behtoui, 2007; Li et al., 2008; McDonald, 2011). However, a recent study looking at students’ social capital did not observe a consistent capital deficit for minorities either (Nygård & Behtoui, 2020). Perhaps differences in access to social capital develop later in life, potentially due to minorities’ lower career success and segregated personal lives, and are less apparent in young people’s social capital. These findings do not support the notion of a capital deficit among minority members, suggesting that a lack of social capital among minority members does not explain labor market inequalities.

The current study builds on previous research by investigating the relationship between social capital and labor market success in a more robust way. We studied early-career success, which reduces the confounding effect of attained skills and social capital during employment (Arulampalam et al., 2001; Gregg, 2001). The role of social capital may have been overestimated in previous studies that looked into the effect of social capital on labor market success at later career stages. Furthermore, a longitudinal method was employed. Social capital was measured at an earlier time point than labor market success for most respondents. This approach reduces the possibility of reverse causality, as social capital may lead to labor market success, but the reverse may also be true (Mouw, 2006). Moreover, we investigated the role of three previously suggested dimensions of social capital on labor market success (Song & Lin, 2009).

Nevertheless, the results of the current study need to be treated with caution. The operationalization of the three dimensions of social capital is a novelty, with which we aimed to capture the composition of the social capital of young adults better than with standard measures and position generators that are typically tailored to adults. However, more research is needed to validate a measure of social capital among youths. The model fit statistics of the current operationalization are suboptimal. Collecting additional data aimed at measuring the three dimensions of social capital may help in constructing a more reliable measure. Alternative operationalizations may also strengthen the finding that minority members score higher on the three dimensions of social capital. Range was, for example, partially measured through the ethnic diversity of one’s network. Future research is needed to investigate whether ethnic minorities also have a higher level of range when an alternative operationalization that does not include ethnic diversity is used.

Another shortcoming of the current study is the potential selectivity of the sample. The majority of the respondents who initially were part of the CILS sample could not be analyzed in the current study. This panel attrition may have led to bias, since individuals lower in socioeconomic status, with more unstable earnings, and with immigration backgrounds are more likely to attrite (Fitzgerald et al., 1998). Excluding economically unsuccessful minorities may be a partial explanation for not finding a social capital deficit among minority members in the current study. Nevertheless, if we thus have studied only the most economically successful minority members, the return deficits they face may be even larger in the full population.

Future research is needed to investigate whether the outcomes of the current study are generalizable to other contexts. The high level of regulation in the Dutch labor market (Steijn et al., 2006) may, for example, affect the returns from social capital. Summarizing, we conclude that young adult minorities face a return deficit on their social capital in the Dutch labor market. We encourage more research into the exact mechanisms behind this finding, using validated measures of social capital of young adults.

## Appendix 1

**Table:** Logistic regression analysis of panel attrition (1 = included in the analysis, 0 = excluded in the analysis) (N = 7331)

	B	*SE*	*OR*
Threshold	− .693	.865	
*Independent variables*			
Minority	− .630**	(.063)	.532
Male	− .473**	(.050)	.623
Age	− .016	(.036)	.984
Parental ISEI	− .005*	(.002)	.995
Education wave 1 and 2^a^	− .130**	(.025)	.878

^a^Four categories (lower pre-vocational education, higher pre-vocational education (vmbo-tl), higher general continued education (havo), and preparatory scientific education (vwo)

## Appendix 2

**Table:** Number of observations, means, and standard deviations of the items used to measure social capital (N = 2574)

Item	Majority members (N = 2076)			Minority members (N = 498)		
	N	Mean	SD	N	Mean	SD
Upper reachability						
Friend’s education	2.031	3.95	.86	467	3.88	.88
*How many people do you know personally*						
Who: are a lawyer?	1.550	.90	2.53	304	2.24	5.72
Who: own a villa?	1.550	2.59	5.37	304	3.15	7.69
Who: are a professor?	1.550	1.25	4.30	304	1.72	5.82
Range						
Educational variety	1.790	2.62	.66	383	2.68	.65
Friends’ ethnic diversity	2.076	2.13	.98	498	3.49	.98
Contacts’ ethnic diversity	1.820	3.02	.90	395	3.77	.86
Extensity						
*How many people do you know personally*						
With the name: Thomas?	1.790	2.39	2.74	383	1.33	3.06
With the name: Kevin?	1.790	2.68	2.93	383	2.26	3.80
With the name: Anne?	1.790	2.70	2.87	383	1.89	3.87
With the name: Melissa?	1.790	2.15	2.84	383	3.10	5.73
With the name: Moham(m)ed?	1.789	1.57	5.02	383	8.20	12.26
Who live in Groningen?	1.786	3.84	10.27	383	1.63	5.60
Who live in Utrecht?	1.784	2.28	5.46	380	4.07	8.32
Who live in Maastricht?	1.782	.90	3.20	382	1.05	4.07
Who live in The Hague?	1.782	2.26	6.76	381	11.19	17.41
Who live in Zwolle?	1.776	1.54	5.07	379	1.33	5.70

## Appendix 3

**Table:** Robustness analysis with unemployment 3 months after graduation as dependent variable (N = 2574)

	Model 0		Model 1		Model 2 majority		Model 2 minority	
	B	*SE*	B	*SE*	B	*SE*	B	*SE*
Threshold	5.217*	1.505	5.130**	(1.444)	7.159**	(2.006)	9.109*	(2.911)
*Independent variables*								
Minority	.028	(.721)	− 1.227	(2.966)	–	–	–	–
Upper reachability	− .039	(.747)	− .597	(.480)	− 3.710*	(1.532)	1.012	(1.123)
Range	1.521	(3.722)	5.269	(7.391)	2.829	(2.267)	9.478	(7.786)
Extensity	− .165	(.174)	− .163*	(.060)	− .250	(.195)	− 1.285	(.938)
*Indirect effects*								
Minority → Upper reachability			.106**	(.020)				
Minority → Range			.421**	(.067)				
Minority → Extensity			1.722**	(.148)				
Minority → Upper reachability → Unemployed	− .063	(.051)						
Minority → Range → Unemployed		2.220	(3.060)					
Minority → Extensity → Unemployed		− .281*	(.112)					
*Control variables*								
Education	− .223**	(.057)	− .221**	(.038)	− .213*	(.069)	− .213*	(.069)
Parental ISEI	− .517	(.372)	− .438	(.286)	− .562	(.511)	− .562	(.511)
Age	.206*	(.063)	.193*	(.060)	.291**	(.081)	.291**	(.081)
Male	− .124	(.096)	− .126	(.095)	− .166	(.128)	− .166	(.128)
*Model fit*								
RMSEA	.063		.060		.059		.059	
CFI	.870		.845		.890		.890	
TLI	.744		.769		.821		.821	
R^2^	.180		.142		.328		–	

The dependent variable is unemployment within 3 months after graduation. All independent, and control variables are allowed to correlate. The loadings of the control variables in model 2a and model 2b are constrained to be equal

**p* < 0.05; ***p* < 0.001

## Appendix 4

**Table:** Robustness analysis with observations clustered in NUTS 3 regions instead of schools (N = 2567)

	Model 0		Model 1		Model 2 majority		Model 2 minority	
	B	*SE*	B	*SE*	B	*SE*	B	*SE*
Threshold	4.008**	1.022	3.515*	(1.190)	5.257**	(1.098)	5.656**	(1.391)
*Independent variables*								
Minority	0.161	(0.682)	− 1.537	(3.599)	–	–	–	–
Upper reachability	− 0.735	(0.875)	− 1.602*	(0.617)	− 2.028	(1.496)	0.645	(0.540)
Range	1.143	(3.558)	5.747	(6.233)	− 0.536	(1.827)	3.282	(2.266)
Extensity	− 0.134	(0.149)	− 0.264**	(0.061)	0.020	(0.186)	− 0.583	(0.338)
*Indirect effects*								
Minority → upper reachability			0.174**	(0.040)				
Minority → range			0.654**	(0.128)				
Minority → extensity			2.628**	(0.294)				
Minority → upper reachability → unemployed	− 0.279*	(0.111)						
Minority → range → unemployed		3.757	(3.596)					
Minority → extensity → unemployed		− 0.694**	(0.158)					
*Control variables*								
Education	− 0.217**	(0.051)	− 0.214**	(0.026)	− 0.264**	(0.049)	− 0.264**	(0.049)
Parental ISEI	− 0.439	(0.239)	− 0.475*	(0.204)	0.005*	(0.493)	0.005*	(0.493)
Age	0.159**	(0.043)	0.135*	(0.047)	0.195**	(0.038)	0.195**	(0.038)
Male	− 0.085	(0.095)	− 0.110	(0.098)	− 0.043	(0.098)	− 0.043	(0.098)
*Model fit*								
RMSEA	0.056		0.047		0.047		0.047	
CFI	0.864		0.872		0.914		0.914	
TLI	0.731		0.809		0.860		0.860	
R^2^	0.176		0.141		0.224		0.056	

The dependent variable is unemployment twelve months after graduation. Based on the NUTS 3 region in which respondents were living during the first wave of data collection. All independent, and control variables are allowed to correlate. The loadings of the control variables in model 2a and model 2b are constrained to be equal

**p* < 0.05; ***p* < 0.001

## Footnotes

1 In this study we follow a classification by Statistics Netherlands (2022). To enhance simplicity and consistency, first, one-and-a-half, and second-generation immigrants with origins in the Global South will be described by the term ‘ethnic minority members’ throughout the article. Other groups are described by the term ‘ethnic majority members’.

2 A robustness analysis in which a three month period was used yielded similar results (Appendix 3).

3 Social capital was measured across all seven waves of data collection. Part of the respondents (N = 737) participated in a wave of data collection after entering the labor market.

4 However, items from different waves are used and response categories are diverse. The fit is well when running separate confirmatory factor analyses for the two reflective latent variables (RMSEA < .01, CFI > .95, and TLI > .95).

5 A non-significant main effect but a significant Wald test was also found in a robustness analysis (Appendix 4) in which observations were clustered in NUTS 3 regions instead of schools (χ^2^(1) = 3.648, *p* = .024).

## References

[CR1] Arulampalam W, Gregg P, Gregory M (2001). Unemployment scarring. The Economic Journal.

[CR2] Aschaffenburg K, Maas I (1997). Cultural and educational careers: The dynamics of socialreproduction. American Sociological Review.

[CR3] Behtoui A (2007). The distribution and return of social capital: Evidence from Sweden. European Societies.

[CR4] Blommaert L, Coenders M, Van Tubergen F (2013). Discrimination of Arabic-named applicants in the Netherlands: An internet-based field experiment examining different phases in online recruitment procedures. Social Forces.

[CR5] Bolt G, Özüekren AS, Phillips D (2010). Linking integration and residential segregation. Journal of Ethnic and Migration Studies.

[CR6] Boudon R (1974). Education, opportunity, and social inequality: Changing prospects in western society.

[CR7] Breen R, Jonsson JO (2005). Inequality of opportunity in comparative perspective: Recent research on educational attainment and social mobility. Annual Review of Sociology.

[CR8] CILS4EU. (2016). *Children of immigrants longitudinal survey in four European countries*. Technical report. Wave 1—2010/2011, v1.2.0. Mannheim University.

[CR9] Caspi A, Wright BRE, Moffitt TE, Silva PA (1998). Early failure in the labor market: Childhood and adolescent predictors of unemployment in the transition to adulthood. American Sociological Review.

[CR10] Corak M (2013). Income inequality, equality of opportunity, and intergenerational mobility. Journal of Economic Perspectives.

[CR11] Erickson BH, Lin N, Cook K, Burt RS (2001). Good networks and good jobs. Social capital: Theory and research.

[CR12] Eurostat. (2016a). *First and second-generation immigrants—Statistics on labor market indicators—Statistics explained*. Retrieved from https://ec.europa.eu/eurostat/statistics-explained/index.php/First_and_second-generation_immigrants_-_statistics_on_labor_market_indicators

[CR13] Eurostat. (2016b). *First and second-generation immigrants—Statistics on employment conditions—Statistics explained*. Retrieved from https://ec.europa.eu/eurostat/statistics-explained/index.php?title=First_and_second-generation_immigrants_-_statistics_on_employment_conditions

[CR14] Fitzgerald J, Gottschalk P, Moffit R (1998). Special issue: Attrition in longitudinal surveys. The Journal of Human Resources.

[CR15] Gallie, D. (1997). *Employment, unemployment and the quality of life: The employment in Europe Survey 1996*. Report prepared for the European Commission.

[CR16] Gregg P (2001). The impact of youth unemployment on adult unemployment in the NCDS. The Economic Journal.

[CR17] Granovetter MS (1973). The strength of weak ties. American Journal of Sociology.

[CR18] Heath AF, Rothon C, Kilpi E (2008). The second generation in Western Europe: Education, unemployment, and occupational attainment. Annual Review of Sociology.

[CR19] Holzer HJ (1987). Informal job search and black youth unemployment. The American Economic Review.

[CR20] Jaspers, E., & Van Tubergen, F. (2014). *Children of Immigrants Longitudinal Survey in the Netherlands (CILSNL)—Wave 4. Reduced version v4.1.0*. DANS.

[CR21] Jaspers, E. & Van Tubergen, F. (2015). *Children of Immigrants Longitudinal Survey in the Netherlands (CILSNL)—Wave 5. Reduced version v5.0.0*. DANS.

[CR22] Jaspers, E., & Van Tubergen, F. (2016). *Children of Immigrants Longitudinal Survey in the Netherlands (CILSNL)—Wave 6. Reduced version v6.0.1*. DANS.

[CR23] Jaspers, E., & Van Tubergen, F. (2017). *Children of Immigrants Longitudinal Survey in the Netherlands (CILSNL)—Wave 7. Reduced version v7.0.1*. DANS.

[CR24] Kalter, F., Heath, A. F., Hewstone, M., Jonsson J. O., Kalmijn, M., Kogan, I., & Van Tubergen, F. (2016a). *Children of Immigrants Longitudinal Survey in Four European Countries (CILS4EU)—Reduced version*. Reduced data file for download and off‐site use. GESIS Data Archive, Cologne, ZA5656 Data file Version 1.2.0. 10.4232/cils4eu.5656.1.2.0

[CR25] Kalter, F., Heath, A. F., Hewstone, M., Jonsson J. O., Kalmijn, M., Kogan, I., & Van Tubergen, F. (2016b). *Children of Immigrants Longitudinal Survey in Four European Countries (CILS4EU)—Reduced version*. Reduced data file for download and off‐site use. GESIS Data Archive, Cologne, ZA5656 Data file Version 2.3.0. 10.4232/cils4eu.5656.2.3.0

[CR26] Kalter, F., Heath, A. F., Hewstone, M., Jonsson J. O., Kalmijn, M., Kogan, I., & Van Tubergen, F. (2016c). *Children of Immigrants Longitudinal Survey in Four European Countries (CILS4EU)—Reduced version*. Reduced data file for download and off‐site use. GESIS Data Archive, Cologne, ZA5656 Data file Version 3.1.0. 10.4232/cils4eu.5656.3.1.0

[CR27] Lai G, Lin N, Leung SY (1998). Network resources, contact resources, and status attainment. Social Networks.

[CR28] Lancee B (2010). The economic returns of immigrants’ bonding and bridging social capital: The case of the Netherlands. International Migration Review.

[CR29] Lancee B (2012). The economic returns of bonding and bridging social capital for immigrant men in Germany. Ethnic and Racial Studies.

[CR30] Lancee B (2016). Job search methods and immigrant earnings: A longitudinal analysis of the role of bridging social capital. Ethnicities.

[CR31] Li Y, Savage M, Warde A (2008). Social mobility and social capital in contemporary Britain. The British Journal of Sociology.

[CR32] Lin N (1999). Social networks and status attainment. Annual Review of Sociology.

[CR33] Lin N (1999). Building a network theory of social capital. Connections.

[CR34] Lin N (2000). Inequality in social capital. Contemporary Sociology.

[CR35] Lin N (2001). Social capital: A theory of social structure and action.

[CR36] Lin N, Dumin M (1986). Access to occupations through social ties. Social Networks.

[CR37] Lin N, Ensel WM, Vaughn JC (1981). Social resources and strength of ties: Structural factors in occupational status attainment. American Sociological Review.

[CR38] Marsden PV (1987). Core discussion networks of Americans. American Sociological Review.

[CR39] Marsden PV (1994). The hiring process: Recruitment methods. American Behavioral Scientist.

[CR40] McCormick TH, Salganik MJ, Zheng T (2010). How many people do you know? Efficiently estimating personal network size. Journal of the American Statistical Association.

[CR41] McDonald S (2011). What's in the “old boys” network? Accessing social capital in gendered and racialized networks. Social Networks.

[CR42] McPherson M, Smith-Lovin L, Cook JM (2001). Birds of a feather: Homophily in social networks. Annual Review of Sociology.

[CR43] Mouw T (2003). Social capital and finding a job: Do contacts matter?. American Sociological Review.

[CR44] Mouw T (2006). Estimating the causal effect of social capital: A review of recent research. Annual Review of Sociology.

[CR45] Nygård O, Behtoui A (2020). Access to social capital and educational returns for children of immigrants: Evidence from three Swedish studies. Nordic Journal of Migration Research.

[CR46] Oaxaca RL, Ransom MR (1994). On discrimination and the decomposition of wage differentials. Journal of Econometrics.

[CR47] Pedulla DS, Pager D (2019). Race and networks in the job search process. American Sociological Review.

[CR48] Pellizzari M (2010). Do friends and relatives really help in getting a good job?. ILR Review.

[CR49] Petersen AM, Jung WS, Yang JS, Stanley HE (2011). Quantitative and empirical demonstration of the Matthew effect in a study of career longevity. Proceedings of the National Academy of Sciences.

[CR50] Reimers CW (1983). Labor market discrimination against Hispanic and black men. The Review of Economics and Statistics.

[CR51] Ruiter S, De Graaf ND (2009). Socio-economic payoffs of voluntary association involvement: A Dutch life course study. European Sociological Review.

[CR52] Smith SS (2005). “Don’t put my name on it”: Social capital activation and job-finding assistance among the black urban poor. American Journal of Sociology.

[CR53] Song L, Lin N (2009). Social capital and health inequality: Evidence from Taiwan. Journal of Health and Social Behavior.

[CR54] Statistics Netherlands. (2021). *Arbeidsdeelname en werkloosheid per maand.* Retrieved from https://opendata.cbs.nl/statline/#/CBS/nl/dataset/80590NED/table?dl=55436

[CR55] Statistics Netherlands. (2022). *Persoon met een niet-westerse migratieachtergrond.* Retrieved from: https://www.cbs.nl/nl-nl/onze-diensten/methoden/begrippen/persoon-met-een-niet-westerse-migratieachtergrond

[CR56] Steijn B, Need A, Gesthuizen M (2006). Well begun, half done? Long-term effects of labor market entry in the Netherlands, 1950–2000. Work, Employment and Society.

[CR57] Vacchiano M, Yepes-Cayuela L, Martí J (2021). The family as (one- or two-step) social capital: Mechanisms of support during labor market transitions. Community, Work & Family.

[CR58] Van der Gaag M, Snijders TA (2005). The Resource Generator: Social capital quantification with concrete items. Social Networks.

[CR59] Van Ours JC, Veenman J (2003). The educational attainment of second-generation immigrants in The Netherland. Journal of Population Economics.

[CR60] Van Ours JC, Veenman J (2004). From parent to child: Early labor market experiences of second- generation immigrants in the Netherlands. De Economist.

[CR61] Van Tubergen F, Völker B (2015). Inequality in access to social capital in the Netherlands. Sociology.

[CR62] Verhaeghe PP, Van der Bracht K, Van de Putte B (2015). Inequalities in social capital and their longitudinal effects on the labour market entry. Social Networks.

[CR63] Völker B, Flap H (1999). Getting ahead in the GDR: Social capital and status attainment under communism. Acta Sociologica.

